# The prevalence of novel periodontal pathogens and bacterial complexes in Stage II generalized periodontitis based on 16S rRNA next generation sequencing

**DOI:** 10.1590/1678-7757-2020-0787

**Published:** 2021-05-17

**Authors:** Salem Abu Fanas, Carel Brigi, Sudhir Rama Varma, Vijay Desai, Abiola Senok, Jovita D'souza

**Affiliations:** 1 Ajman University College of Dentistry Department of Clinical Sciences Ajman United Arab Emirates Ajman University, College of Dentistry, Department of Clinical Sciences, Ajman, United Arab Emirates; Center of Medical and Bio-allied Health Sciences research, Ajman University, Ajman, UAE.; Ajman University Center of Medical and Bio-allied Health Sciences research Ajman UAE Ajman University, College of Dentistry, Department of Clinical Sciences, Ajman, United Arab Emirates; Center of Medical and Bio-allied Health Sciences research, Ajman University, Ajman, UAE.; 2 Ajman University College of Dentistry Department of General Dentistry Ajman United Arab Emirates Ajman University, College of Dentistry, Department of General Dentistry, Ajman, United Arab Emirates.; 3 Mohammed Bin Rashid University of Medicine and Health Sciences College of Medicine Department of Microbiology UAE Mohammed Bin Rashid University of Medicine and Health Sciences, College of Medicine, Department of Microbiology.; 4 Gulf Medical University College of Dentistry Department of Periodontics Ajman UAE Gulf Medical University, Department of Periodontics, College of Dentistry, Ajman, UAE.

**Keywords:** Periodontal pathogen, 16S rRNA, Next-Generation sequencing, Microbial profile

## Abstract

**Objective:**

To define the subgingival microbial profile associated with Stage II generalized periodontitis using next-generation sequencing and to determine the relative abundance of novel periodontal pathogens and bacterial complexes.

**Methodology:**

Subgingival biofilm samples were collected from 80 subjects diagnosed with Stage II generalized periodontitis. Bacterial DNA was extracted, and 16S rRNA-based bacterial profiling via next-generation sequencing was carried out. The bacterial composition and diversity of microbial communities based on the age and sex of the patients were analyzed. The bacterial species were organized into groups: bacterial complexes (red, orange, purple, yellow, and green), novel periodontal pathogens, periodontal health-related species, and unclassified periodontal species. The results were analyzed and statistically evaluated.

**Results:**

The highest number of bacteria belonged to the phylum Bacteroidetes and Firmicutes. In terms of relative abundance, the orange complex represented 18.99%, novel bacterial species (*Fretibacterium* spp. and *Saccharibacteria* spp.) comprised 17.34%, periodontal health-related species accounted for 16.75% and unclassified periodontal species represented (*Leptotrichia* spp. and *Selenomonas* spp.) 15.61%. Novel periodontal pathogens had outweighed the periodontal disease-related red complex (5.3%). The one-sample z-test performed was statistically significant at p<0.05. The Beta diversity based on the unweighted UniFrac distance at the species level demonstrated a total variance of 15.77% based on age and 39.19% on sex, which was not statistically significant.

**Conclusion:**

The bacterial species corresponding to the disease-related orange complex and novel periodontal pathogens are predominant in Stage II generalized periodontitis.

## Introduction

Periodontitis is a biofilm-associated inflammatory disease of the periodontium characterized by the destruction of periodontal tissue. Three significant factors are involved in the pathogenesis of periodontitis: susceptible host, presence of periodontal pathogens, and reduction or absence of beneficial bacterial species for periodontal health. Whilst, the microbial composition of subgingival biofilm differs in healthy individuals, the development of periodontitis is directly co-related to a characteristic shift in the microbiome referred to as “Dysbiosis.”^1^^,^^2^ The inter-relationship between dysbiosis of the oral microbiome and the periodontitis progression has been established by several studies.^3^^,^^4^ These investigations postulate the role of the microbiome on the onset and progression of periodontitis.

The role of microbiome relating to periodontal health and disease was first described by Socransky, et al.^5^ (1998). This path-breaking study – using whole genomic DNA probes to 40 bacterial species – has defined the potential role of the bacterial complex as opposed to individual bacterial species. The red complex is composed of three bacterial species: *Treponema denticola, Porphyromonas gingivalis*, and *Tannerella forsythia* that were associated with periodontal disease. The yellow complex composed of different *Streptococcus* species and green complex composed of *Capnocytophaga* species were firmly related to periodontal health. The orange complex – including *Fusobacteria* species – members of *Prevotella*, and *Campylobacter* were classified as periodontopathogens. The detection and quantitation of these bacterial complexes were described with whole genomic DNA probes, checkerboard DNA-DNA hybridization,^5^ and using highly sensitive techniques such as real-time PCR (polymerase chain reaction).^6^ These test may also be used to quantify species from Socransky complexes.

However, recent investigations using metagenomics and metatranscriptomics proposed that more diverse periodontitis-associated microbiota are involved in periodontal disease. According to this model, periodontitis results from polymicrobial synergy and dysbiosis that disturbs the ecologically balanced biofilm associated with periodontal tissue homeostasis.^7^ In this model, the host's immune response is initially disrupted by keystone pathogens supported by accessory pathogens and it is subsequently over-activated by pathobionts resulting in homeostasis breakdown and destructive inflammation. The host's immune response is dysregulated either because it is disrupted by the microbial community or due to host immunoregulatory defect, resulting in bacterial outgrowth and overt pathogenicity.^8^

Recent studies have also identified novel periodontal pathogens and additional bacterial species associated with periodontal health and disease. A study conducted by Kirst, et al.^9^ (2015) has confirmed the presence of *Rothia* and *Streptococcus* species related to periodontal health. Similarly, investigations based on next-generation sequencing has revealed the presence of novel periodontal pathogens/pathobionts like^10^^,^^11^^,^^12^ *Fretibacteirum fastidiosum, Filifactor alocis*, and *Eubacterium saphenum*. A recent review of the role of bacterial biofilm in periodontal diseases has highlighted the presence of novel pathogens and health-related species in the subgingival biofilm. The review based on next generation sequencing studies has categorized *Fretibacterium* spp., *Saccharibacteria* spp., *Dialister* spp., as novel pathogens and *Rothia* spp., *Kingella* spp., *Gemella* spp., *Corynebacterium* spp. as health-related species.^13^

Despite some studies revealing the presence of novel bacterial species, evidence reporting their relative prevalence regarding established bacterial complexes are rare. 16S rRNA-based bacterial method constitute an efficient method to determine the composition and diversity of bacterial communities.^14^ Therefore, this study aims to illustrate the subgingival bacterial profile associated with Stage II generalized periodontitis using NGS of 16S RNA gene, and to identify the relative abundance of novel periodontal pathogens and bacterial complexes.

## Methodology

### Study population

This investigation was developed in compliance with the Helsinki Declaration on Medical Research Involving the Ethics Committee at Ajman University (F-H17-11-03). The study was registered under clinical trial with the identification number as NCT04425343. Prior to the participation in the study, informed consent from all the participants was obtained. The study was conducted from September 2018 to November 2019. The included participants had no history of systemic diseases (hypertension, diabetes mellitus, etc.) and they had not received any antibiotics three months prior to sampling. Exclusion criteria included pregnancy, history of smoking, and periodontal therapy over the past three months. Patients recruited for the study were diagnosed as Stage II Generalized periodontitis according to the classification defined at the 2017 world workshop: “classification of periodontal and peri-implant disease and conditions.”^15^ Periodontal status presenting a severity of interdental clinical attachment loss of 3-4 mm, radiographic bone loss between 15%-33%, and subjects with absence of tooth loss were included. Patients with periodontal pocket of ≤ 5mm with horizontal bone loss and having an extent and distribution with >30% teeth involved were selected. A total of 80 subgingival biofilm samples were collected from 36 females and 44 males, aged from 25 to 39 years. Among the total participants, 28 patients (12 females; 16 males) were in the age group of 25-32yrs and 52 patients (24 females; 28 males) aged from 33 to 39yrs. The buccal aspect of maxillary molars and mandibular molars were isolated with cotton rolls and air dried gently to discard the supragingival plaque with curettes. Using a sterile Gracey curette no 9-10, 11-12 (Hu-Friedy, USA), subgingival biofilm was removed from the base of periodontal pocket with slight pressure and collected to the level of 0.1 mL in 1.5 mL microcentrifuge tube containing 200µL of sterile Buffer CL (ComplexioLyte buffer - for solubilization of membrane proteins and protein complexes in bacteria).

### DNA extraction, library construction, sequencing, and data processing

DNA extraction was carried out using the QIAmp^®^ DNA (Qiagen, Hilden, Germany) extraction kit according to the manufacturer's guidelines. After measuring DNA purity, the amount of DNA per sample was quantified using the DeNovix dsDNA Broad Range Assay kit (DeNovix, Wilmington DE, USA), and samples had a concentration of 36±14 ng/µL for downstream workflow. For next generation sequencing, we amplified the V3-V4 region of the 16S rRNA gene using the Taq PCR Master Mix Kit (Qiagen). The full-length primer sequences, using standard IUPAC nucleotide nomenclature, to follow the protocol targeting this region were:

16SAmpliconPCRForwardPrimer=5'
TCGTCGGCAGCGTCAGATGTGTATAAGAGA CAGCCTACGGGNGGCWGCAG
16SAmpliconPCRReversePrimer=5'
GTCTCGTGGGCTCGGAGATGTGTATAAGAGA CAGGACTACHVGGGTATCTAATCC

**Figure 1:**
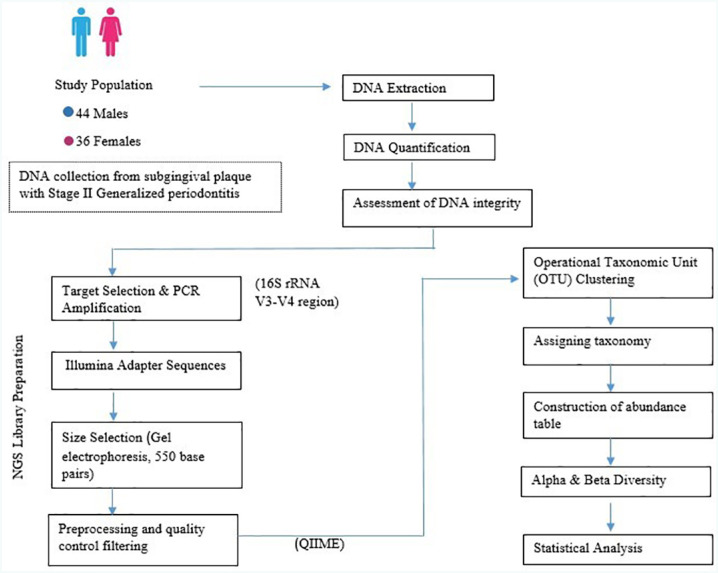
Experimental design and workflow for microbial profiling in samples diagnosed as Stage II generalized periodontitis

Aliquots of the PCR product were assessed on the Fragment Analyzer (Advanced Analytical, Parkersburg WV, USA) and purified using Agencourt AMPure XP beads (Beckman Coulter, Atlanta GA, USA) and quantified using the DeNovix dsDNA High Sensitivity Assay kit (DeNovix) following manufacturer's protocols. Unique oligonucleotide indexes were added to the amplicons using the Nextera XT Index Kit (Illumina, San Diego, USA), indexed amplicons were purified using Agencourt AMPure XP beads (Beckman Coulter) and fragment lengths were assessed on the Fragment Analyzer using High Sensitivity Small Fragment Analysis Kit (Advanced Analytical). Up to 96 sequencing libraries with unique Nextera XT Indexes were pooled in equimolar concentrations in a single reaction tube and sequenced on the MiSeq instrument (Illumina) using the MiSeq Reagent 600 cycle v3 kit (Illumina). The bioinformatics pipeline used for processing microbiome 16S sequence data was QIIME (Quantitative Insights into Microbial Ecology). The bioinformatics pipeline consisted of five main steps, as described in Figure 1. Sequences shorter than 200 bp, indicating a Phred score lower than 20 were removed using the QIIME2 dada2 denoise-paired module. The remaining sequence data was used for de novo binning of operational taxonomic units (OTUs) based on sequence similarity followed by selection of representative OTUs per bin. All the clean tags from 80 samples were clustered into OTUs using QIIME at 97% sequence similarity. The 16S Metagenomics workflow classified organisms based on V3 and V4 amplicon utilizing a database of 16S rRNA data. Classification of reads at several taxonomic levels was based on Greengenes database (http://greengenes.lbl.gov/). Multiple sequence alignment of representative sequences and microbial community profiling was done using QIIME2. Estimation of alpha and beta diversity was carried out based on the operational taxonomic units.

Bacterial species were sorted into groups in order to obtain the relative abundance of bacterial complexes and novel periodontal pathogens. The data collected with QIIME analysis at level seven was classified into their group, and the bacterial species that had no established role in periodontitis and prevalence inferior to 0.01% were excluded. The species were classified based on their bacterial complexes (red, orange, purple, yellow, and green), novel periodontal pathogens, health-related species, and health/disease species.^5^^,^^13^ Health/disease species group were neither categorized as health nor disease species due to the lack of evidence supporting their role as periodontal pathogens/health species.^13^^,^^16^ The percentage of total abundance in each group was estimated by combining the abundance of individual species in each group. The relative abundance of each group for all subgingival biofilm samples was graphically plotted.

### Statistical analysis

The data were entered using Microsoft Excel 2016 and imported into IBM SPSS Statistics 26 (IBM Corporation, New York, NY, United States) for statistical analysis. For investigating the means for different phylum and bacterial complexes a one-sample t-test was performed at p<0.05. Furthermore, all quantitative variables related to clinical parameters were determined using Levene's Test for equality of variances. The evaluation of microbial community among all the biofilm samples for similarity was determined using Principal coordinates analysis (PCoA) performed at the species level. The intra- and inter-group similarity based on the Unweighted UniFrac distance – which represents the phylogenetic measurement of beta diversity – was estimated using QIIME. The Spearman's correlation coefficient was used to analyze the difference between the Unweighted UniFrac distances among two groups (based on the age and sex) and significance was set at p<0.05.

## Results

### Sequencing

**Figure 2:**
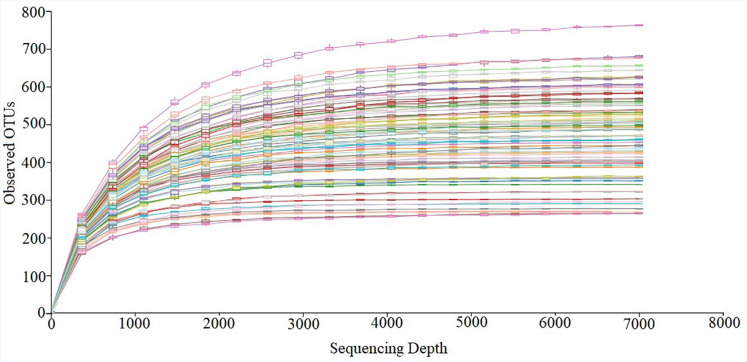
Represents rarefaction curves for the number of operational taxonomic units in each sample. Each sample received a separate color code representing its corresponding number of OTUs. The rarefaction curve based on 97% similarity were each color code given to 80 samples

The Illumina MiSeq sequencing reinstated a total of 9,316,880 filtered clean tags, with an average of 114,461±2,570 sequences per sample. The OTU richness varied from 271 to 580 per sample. Figure 2 presents rarefaction curves showing the observed OTU richness (97% sequence similarity as cutoﬀ) with increasing sequence depth. All subgingival biofilm samples have attained a plateau, as depicted in the graph by the rarefaction curves obtained from OTUs. The asymptotic curves expressed in Figure 2 revealed that the sampling quality was excellent, indicating the probability that the addition of new species to the final samples has reached zero.

### Microbial taxonomic composition

**Figure 3:**
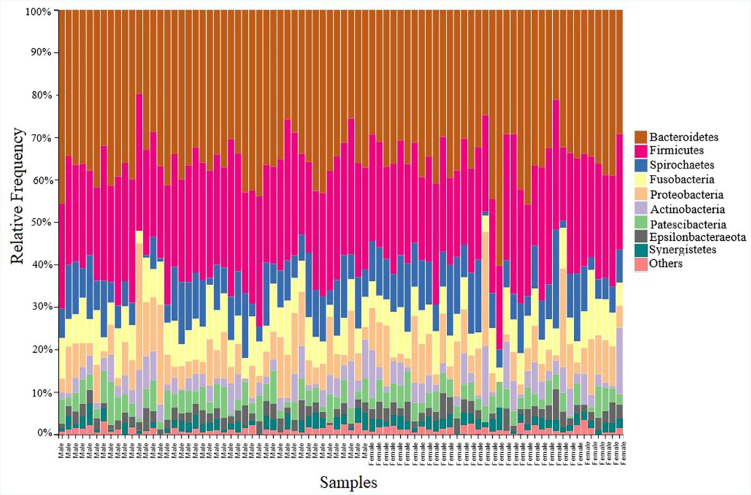
The relative frequency of the taxonomic composition in all the 80 sub-gingival biofilm samples. The bars are organized according to patients’ sex. The group mentioned as others refer to the phylum presenting values below 1%

Figure 3 depicts nine phyla, which were commonly present in all samples diagnosed as Stage II generalized periodontitis. The phylum of Bacteroidetes exhibited a high prevalence, followed by Firmicutes representing 34.15% and 25.14%, respectively. A statistically significant result in the mean scores for Bacteroidetes (M=34.15±5.3) and for Firmicutes (M=25.14±3.8) was observed at conditions t (79) =57.055 p<0.05, t (79)=59.14, p<0.05 respectively. The phylum including Spirochetes 8.60%, Fusobacteria 7.50%, Proteobacteria 7.33%, and Actinobacteria 6.18% represented a similar frequency. Statistically significant results were also obtained for Spirochetes (M=8.60±4.3) at t (79)=17.681; Fusobacteria (M=7.50±2.55) at t (79)=26.24; Proteobacteria (M=7.33±5.1) at t (79)=13.01 and Actinobacteria (M=6.18±3.8) at t (79)=12.50 for p<0.05. The remaining phyla consisting of Patescibacteria 3.95%, Epsilonbacteraeota 2.65%, and Synergistetes 1.7% constituted the minority with their statistical significant figures being (M=3.95±1.8) at t (79)=19.26; (M=2.65±1.2) at t (79)=18.75; (M=1.95±0.8) at t (79)=20.47 at p>0.05 respectively. The phyla group mentioned as “others” consisted of Chloroflexi, Elusimicrobia, and Verrucomicrobia. The species of bacteria in these groups of phyla were not considered in this study as they were not consistently present in all samples, also, they represented a frequency inferior to 1%. Levene's test for equality of variances for our analysis was F (2,78)=1.49, p=0.226. Since the significance was greater than 0.05, the Levene's Test was non-significant, and homogeneity of variance at phylum level were observed for age and sex.

### Prevalence of bacterial class in stage II generalized periodontitis

**Figure 4:**
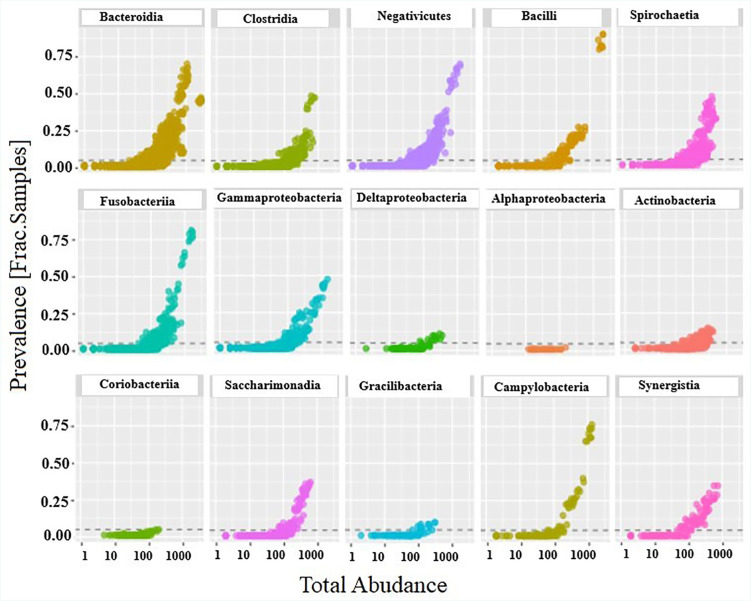
The prevalence of each bacterial class as fraction per sample plotted against their total abundance

**Figure 5:**
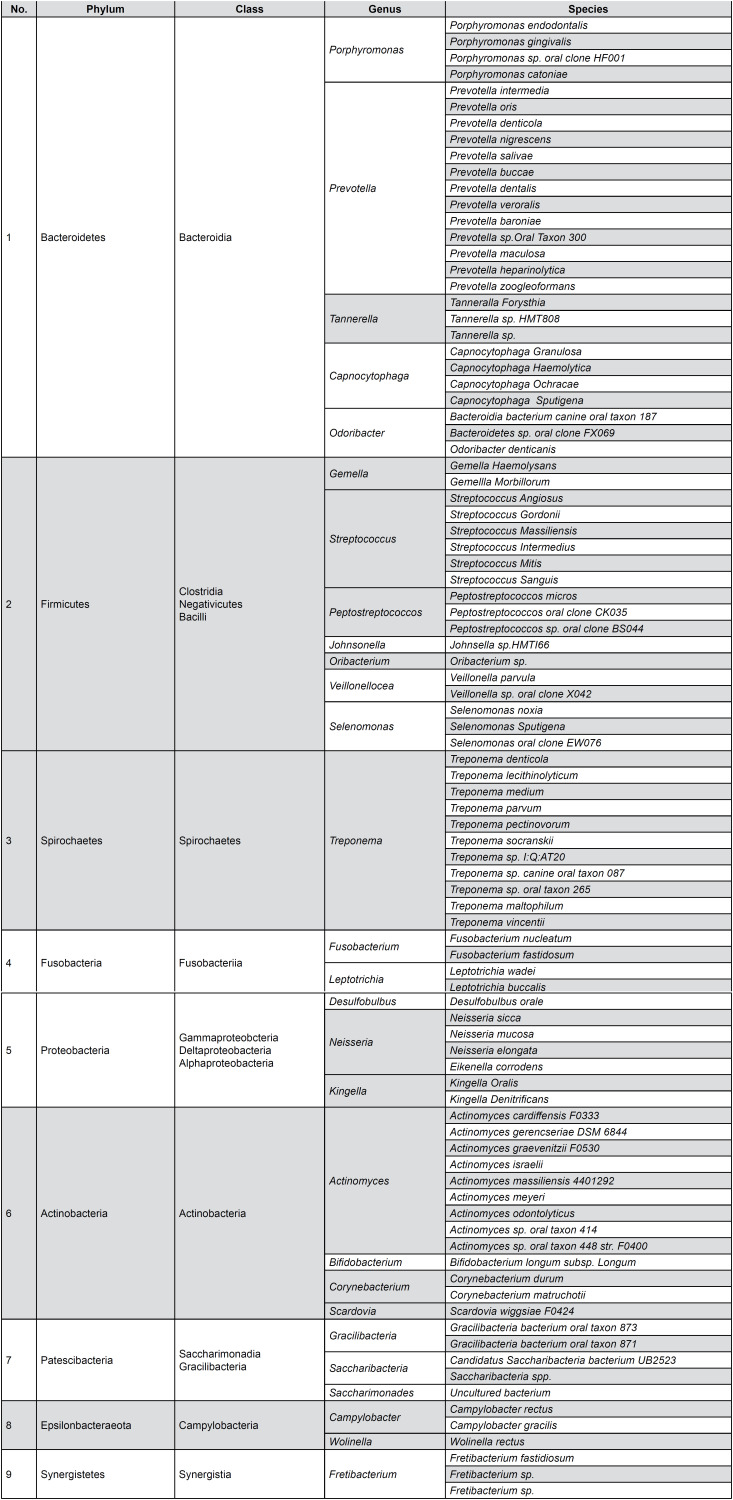
Bacterial species present in all the biofilm samples and their corresponding genus, class, and phylum

The relative abundance of the different classes of bacteria were analyzed by computing scatter plots. The X-axis in the graph representes the total count of bacteria in each class and Y-axis their corresponding prevalence as fraction per samples. The correlation between presence of these bacterial classes and their incidence in Stage II periodontitis, was analyzed. All 15 bacterial classes presented tight clusters in an upward pattern curve (Figure 4). The tighter clustering represented a stronger relationship between the presence of each bacterial class in all samples diagnosed as Stage II periodontitis. Outliers were seen in case of Bacilli, Clostridia, and Bacteroidia but were relatively insignificant. The most prevalent class of bacteria per sample were Bacteroidia, Negativicutes, Spirochaetes, and Fusobacteria. The least predominant were Gracilibacteria, Coriobacteria, and Alphaproteobacteria bacterial class representing a frequency of less than 0.25 fraction per sample. The phyla, class, genus, and species isolated from the subgingival biofilm of all 80 subjects diagnosed as Stage II generalized periodontitis are shown in Figure 5.

### Abudance of microbial complexes and novel bacterial species

**Figure 6:**
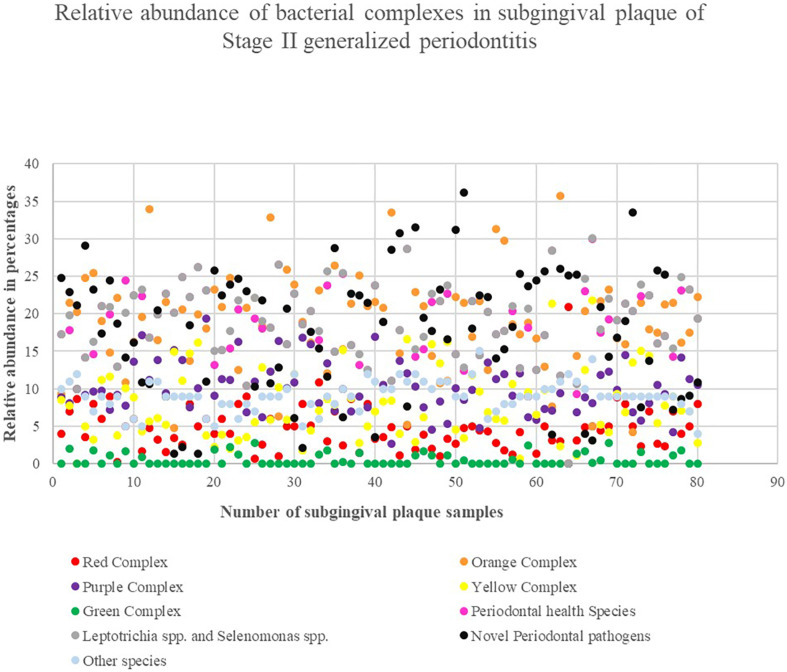
The relative abundance of Socransky's bacterial complex and novel bacterial species. The X-axis represents the number of plaque samples, and Y-axis depicts the corresponding percentages

Table 1 reveals the data obtained for the relative abundance of bacterial species sorted into nine groups. The one-sample z-test performed for each group was statistically significant at p<0.05. The t statistics results are described in Table 1. The periodontal disease species included for novel periodontal pathogens were *Fretibacterium* spp. and *Saccharibacteria* spp. The health-related species was composed of *Neisseria* spp., *Gemella* spp., *Rothia* spp., *Kingella* spp., and *Corynebacterium* spp. The group composed of *Leptotrichia* and *Selenomonas* species represented 15.61%. The red complex composed of T. *denticola*, T. *forsthyia* and *P. gingivalis* represented a relative frequency of 5.3%. The orange complex, novel-periodontal pathogen, and periodontal health-species indicated a significant value of 18.99%, 17.34%, and 15.61%, respectively. Among the health-related complexes, the green complex (1.33%) was minimally present compared to the yellow (7.49%) and purple (7.9%) complexes. The relative abundance of Socransky's bacterial complexes and novel bacterial species in all samples are depicted in Figure 6.

**Table 1:** The relative abundance of bacterial species from level 7 categorized into subgingival microbial complexes

No	Bacterial Complex	Bacterial Species	Relative Abundance in %	Total abundance in Percentages	T statistics	P value
1	Red Complex	*T. Denticola* †	2,95	5,30%	12,06	0,015
		*P. Gingivalis*	1,64			
		*T. Forsythia*	0,71			
2	Orange Complex	*Campylobacter Rectus*	1,52	18,99%	20,37	0,002
		*Fusobacterium Nucleatum*†	11,61			
		*P.micros*	0,82			
		*P.intermedia*	5,04			
3	Purple Complex	*Actinomyces spp.*	3,1	7,90%	24,35	0,002
		*Veillonella spp.*	4,8			
4	Yellow Complex	*S. Gordonii*	1,93	7,49%	14,67	0,001
		*S. Intermedius*	1,81			
		*S. Mitis*	2,03			
		*S. Sanguis*	1,72			
5	Green Complex	*Capnocytophaga spp.*	1,27	1,33%	5,57	0,004
		*E.corrodens*	0,06			
6	Health-related Species	*Neisseria spp.*	5,53	16,75%	32,15	0,002
		*Gemella spp.*	3,31			
		*Rothia spp.*	3,14			
		*Kingella spp.*	2,11			
		*Corynebacterium spp.*	2,66			
7	Novel periodontal pathogens	*Fretibacterium spp.*†	11,81	17,34%	14,06	0,001
		*Saccharibacteria spp.*	5,53			
8	Health/Disease-related	*Leptotrichia spp.*†	8,53	15,61%	32,74	0,007
		*Selenomonas spp.*	7,08			
9	‡Other Species	*Porphyromonas spp.*	2,65	9,29%	16,35	0,023
		*Prevotella spp.*	2,25			
		*Treponema spp.*	2,85			
		*Odoribacter spp.*	0,25			
		*Johnsonella spp.*	0,07			
		*Oribacterium spp.*	0,02			
		*Desulfobulbus*	0,23			
		*A. Actinomycetemcomitans*	0,91			
		*Wolinella spp.*	0,06			

The total abundance in each group was calculated by taking the sum of relative abundance of each bacterial species.

† represents the highest bacterial species in each group.

‡ species that has been excluded in the remaining eight groups and their corresponding prevalence indicated greater than 0.1%.

### Alpha and beta diversity

**Figure 7:**
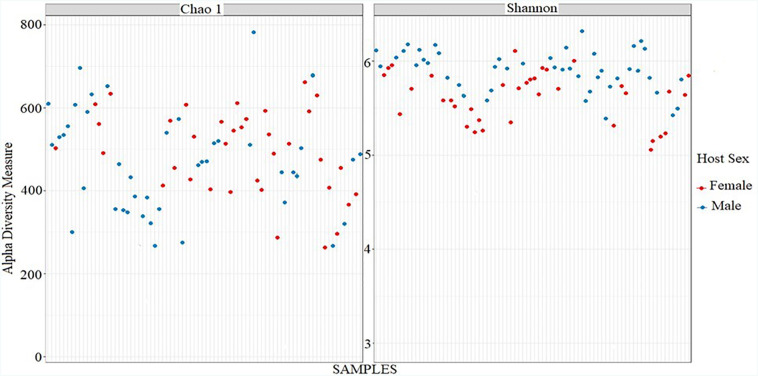
Alpha diversity metrics (a) Chao1 and (b) Shannon Index

Chao^1^ and Shannon were used as alpha diversity metrics to measure the composition of bacterial communities (Figure 7). The plot was generated by taking the total number of OTUs in a sample by using shiny-phyloseq. Figure 7a represents the alpha diversity abundance value of all 80 biofilm samples. The horizontal X-axis is represented by the sample indicated by faint gray lines. Every sample has a separate discrete position on the graph corresponding to the number of OTUs present in that sample. The Y-axis represents the number of OTUs, ranging from 200 to 800 values. The Chao^1^ richness estimate had no statistically significant difference between samples. A total of 42,560 OTUs were found across all biofilm samples. The species evenness was estimated using Shannon diversity index, with the horizontal X-axis representing the number of samples. A Shannon index (Figure 7b) ranging from 5.18 to 6.48 was observed overall, and clustering was detected, indicating an evenness of the bacterial community in all samples.

**Figure 8:**
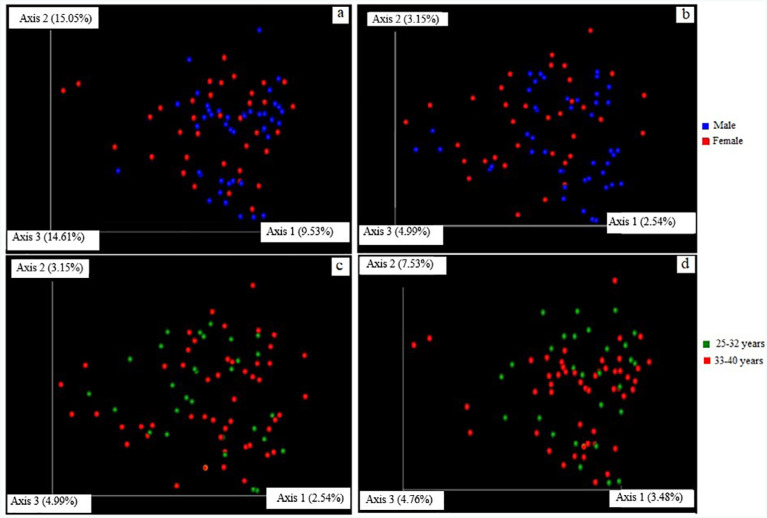
Principal coordinates analysis (PCoA) based on weighted UniFrac distances (a & d) and Jaccard index (b & c). PCoA score plot represents the phylogenetic relationship between biofilm samples diagnosed with Stage II generalized periodontitis according to patients’ sex (a & b) and age (c & d). Axis 1, 2, and 3 are principle coordinates- PC1, PC2, and PC3, respectively

To determine the difference of dissimilarity in microbial complexes at the species level, the weighted UniFrac distance and Jaccard Index were estimated using QIIME. The principal coordinate analysis estimated the distance between samples and presented them into three visual axes based on the variability between samples. The PCO graphs for each sample are represented by a dot based on their UniFrac distance in the 3D graph and the differential coloring are given for sex (blue and red) and age (green and red) characteristics. Clustering was observed in both age and sex due to the samples containing bacterial community of similar composition. A total variance of 39.19% (rs=0.36, P>0.05) was observed for sex (weighted UniFrac) with the first, second, and third axis variability explaining 9.53%, 15.05%, and 14.61% respectively (Figure 8a). Regarding age (weighted UniFrac) 15.77% (rs=0.28, P>0.05) variation was observed with the first, second, and third axis variability explaining 3.48%, 7.53%, and 4.76%, respectively (Figure 8d). The percentage measurement of dissimilarity between the age and sex for all samples were also measured using Jaccard Index. The percentage of variation was calculated to be 10.69% and the Jaccard distance was obtained as 0.89. Therefore, a Jaccard index of 0.89 stated an 89% similarity for two categories of age and sex in Figures 8b and c. The results indicated no statistically significant variation between age and sex across all the 80 biofilm samples.

## Discussion

An imbalance in the dynamic relationships among biofilm, host, and microenvironment results in periodontal diseases.^13^ According to the current classification, Stage I periodontitis indicates borderland between gingivitis and periodontitis, Stage II represents established periodontitis with absence of tooth loss and no significant damage to attachment apparatus.^15^ As the underlying cause of periodontitis is predominantly microbial,^17^ enumerating the subgingival microbial profile of established periodontitis as in Stage II generalized periodontitis would aid us in deeper understanding of initiation and progression of periodontitis. Stage III and Stage IV periodontitis results in tooth loss and significant damage to the attachment apparatus,^15^ hence the relative abundance of all bacterial healthy/disease complexes to novel bacterial species may not be accomplished. Since the grade modifiers for Grade A periodontitis are non-smokers and normoglycemic individuals,^15^ patients with a history of smoking and any systemic illness, were excluded from this study. Participants younger than 40 years were included in the study, as advanced periodontal destruction and bone loss are rarely seen in such individuals.^18^

The most predominant phyla observed in the study relating to Stage II generalized periodontitis were Bacteroidetes and Firmicutes. These outcomes were consistent with the previous investigation conducted by Chen, et al.^16^ (2018) on microbial compositional analysis of periodontal diseases. *Prevotella* and *Porphyromonas* genera detected in our study represents Bacteroidetes phyla which are Gram-negative facultative anaerobes. They are involved in the formation and progression of several forms of periodontal disease and their high number indicates active periodontal disease. *Streptococcus* and *Gemella* genera identified in this study were composed of Gram-positive facultative anaerobes and are classified as firmicutes phyla primarily responsible for periodontal health. Following the above stated phyla, *Spirochetes* and *Fusobacteria* indicated a relatively similar frequency in the entire biofilm samples. The most recognized species in *Fusobacteria* phylum concerning periodontitis is *F. nucleatum*; being prominent quantitatively and the first Gram-negative species to become established in plaque biofilms.^19^ A higher presentation of *F. nucleatum* was observed in this study regarding the orange complex. Similarly, the spirochetal accumulation in subgingival plaque is a function of the clinical severity in periodontal disease.^20^ *T. denticola* which has been established by several studies as disease-related complex represents Spirochetes phylum. In our study *T. denticola* outweighed the other bacterial species (*P. gingivalis* and *Tannerella forsythia*) in the red complex, indicating a direct co-relation to significantly higher Spirochetes phyla. This observation is in agreement with other studies relating to microbial profiles of periodontal disease.^21^^,^^22^

The accompanying phyla identified in Stage II periodontitis along with the above-stated ones, were Proteobacteria, Actinobacteria, Patescibacteria, Epsilonbacteraeota, Synergistetes, and Choloroflexi. In this study, we identified proteobacteria phylum, and their presence was related to periodontal health. *Neisseria* spp. and *Kingella* spp. relating to periodontal health were classified under Proteobacteria phylum. Concerning Actinobacteria phyla, *Actinomyces* spp., *Bifidobacterium* spp., and *Corynebacterium* spp., observed in our study were also related to periodontal health. Actinomyces classified under purple complex are related to periodontal health,^23^ Bifidobacterium are involved in preventing periodontal disease^24^ and Corynebacterium are included as health-related species.^13^ The prevalence of Proteobacteria and Actinobacteria phyla was comparable to the studies conducted by Shi, et al.^25^ (2018) and Chen, et al.^16^ (2018), on subgingival plaque microbial profiling. The statistically significant presence of these periodontal health-related bacterial species that prefer an anaerobic environment indicates its presence in the subgingival plaque of Stage II periodontitis.

Patescibacteria – which is present in our study – was proposed by Rinke, et al.^26^ (2013) as a superphylum composed of Gracilibacteria, Microgenomates, and Parcubacteria. In our study we detected a positive co-relation between Gracilibacteria class and Stage II periodontitis. Gracilibacteria was first described in the human oral microbiome in 2014 with a 16S rRNA screening, but its relation to periodontitis is still unknown.^27^ Epsilonbacteraeota is a relatively new phyla^28^ – which was previously described as a class of Proteobacteria phylum. Epsilonbacteraeota phyla identified in our study consisted of *Wolinella* spp. and *Campylobacter* spp. in the subgingival plaque. Certain *Wolinella* spp. like *Wolinella* rectus are associated with adult periodontitis but their role in the disease remains undefined.^29^ *C. rectus* – which belongs to orange complex – has been detected in our study and the high prevalence of Campylobacter class in fraction per sample was also confirmed. The presence of Synergistetes phyla identified in our research – presenting a stronger association with periodontitis – has been established in investigations conducted by Kirst, et al.^9^ (2015) and Marchesan, et al.^30^ (2015). They have proteolytic properties and are strictly anaerobic bacteria – representing the profiles of periodontal pathogens. Synergistetes species have been associated with gingival bleeding and they prefer a habitat with inflammation and bleeding.^31^ A significant observation in our study was the frequency of Chloroflexi less than 1% in all the 80 subgingival biofilm samples. These findings contrast with observations in a study conducted by Kirst, et al.^9^ (2015), where these authors detected a relatively high prevalence compared with our study. The suggested cause might be due to the selection of cases involved in the study. This study had taken clinical attachment loss (CAL) of 3-4 mm, whereas their study involved CAL of ≥5mm. The presence of all these anaerobic genera that are mentioned from the phyla Firmicutes, Bacteroidetes, Proteobacteria, Spirochaetes, and Synergistetes constitute a dysbiotic microbial community and these findings were consistent with the current literature.^8^^,^^12^^,^^13^

A species-level of identification relating to their representative bacterial complex were described in order to recognize their prevalence in Stage II generalized periodontitis. The orange complex, health-related species, unclassified health/disease periodontal species, and novel periodontal pathogens represented a higher count, which was statistically significant. This was followed by purple, yellow, red, and green complexes. The health-related species (*Neisseria* spp., *Rothia* spp., *Kingella* spp., and *Corynebacterium* spp.) and novel species (*Fretibacterium* spp. and *Saccharibacteria* spp.) has outnumbered the red complex. A similar observation of the red complex consisting of a relatively small fraction of the total bacterial species was established in a study conducted by Kumar, et al.^32^ (2005). A quantitative 16S clonal analysis of periodontal pathogens and beneficial species in their study has detected *Campylobacter, Abiotrophia*, *Gemella, Capnocytophaga*, and *Neisseria* genera outnumbering periodontopathogens. Likewise, the presence of a high proportion of putative and novel pathogens in periodontitis has been established by Colombo, et al.^13^ (2019). All these findings propose that the bacterial species belonging to *Fretibacterium, Saccharibacteria* and other health-related species – that are not included in bacterial complexes – play a pivotal role in the dysbiosis of periodontal disease. Though the presence of red complex bacteria is seen in diminished numbers compared to periodontal health contributing species, the presence of clinical attachment loss of 4 mm could state a foundation for dysbiosis activity in Stage II periodontitis.

The occurrence of all microbial complexes in periodontitis observed in this study constitute an established dysbiosis.^13^ The subgingival plaque composing the microbial complex formed, resembles the sequence of plaque development. The primary colonizers are bacterial species representing the yellow complex. The yellow complex – predominantly composed of *Streptococcus* species – recognizes the complementary salivary receptors in the acquired pellicle (present on tooth) and provides receptors for secondary colonizers. The secondary colonizers include members of the purple and green complex – *Actinomyces naeslundii, Capnocytophaga ochracea*, *Eikenella corrodens*, and *Veillonella atypica*. The primary and secondary colonizers – including yellow, purple and green complexes – are the early colonizers.^13^ Succeeding the growth of early colonizers, *F. nucleatum* colonizes – acting as central species in physical interactions between Gram-positive and Gram-negative species. They act as intermediate colonizers, providing conditions necessary for the emergence of oxygen-intolerant anaerobes.^19^ The red complex considered as the climax community occurring at the final stage uses the anaerobic environment. Thus, during the plaque development, a host-compatible symbiotic relationship gets involved into incipient dysbiotic microflora (gingivitis) leading to a self-resolving inflammatory response. This would further evolve into frank dysbiosis (periodontitis) microflora with ineffective inflammatory responses leading to periodontal tissue destruction. Hence, the presence of bacterial species representing early, intermediate, and climax communities detected in our study, states an established dysbiosis in Stage II periodontitis.

The two novel bacterial species detected in our study were the *Fretibacterium* spp. and *Saccharibacteria* spp. The *Fretibacterium* spp. showed significantly increased levels in the periodontitis cases and were positively correlated with ≥ 4 mm periodontal pocket depth (PPD) and bleeding on probing (BOP).^33^ As these bacterial species could not be cultivated, their characteristics and virulent aspects relating to the disease progression is unknown. The presence of *Fretibacterium* was also confirmed in the subgingival plaque of early stages of chronic periodontitis patients.^34^ *Saccharibacteria* has dynamic interaction with its host bacteria and exhibits virulent killing of host bacterium. These bacteria can inhibit the host's growth dynamics and affect the oral microbial ecology.^35^ *Saccharibacteria* previously known as TM7 detected in our study were also described by Kirst, et al.^9^ (2015) in periodontitis cases. The role of novel periodontal pathogens has been reported.^32^ These pathogens have shown a strong association with periodontal disease compared to less numerous periodontal pathogens previously implicated.

In addition to the novel species, other relevant species on our study were *Neissera* spp. *Gemella* spp., *Rothia* spp. *Kingella* spp., and *Corynebacterium* spp. These bacterial species were clumped together as health-related species based on the review conducted by Colombo, et al.^13^ (2019). *Neissera* spp. and *Gemella* spp., are the microbial profile of periodontal health and they were confirmed as beneficial species with quantitative 16S Clonal Analysis.^32^ The role of *Kingella, Neissera*, and *Gemella* genera in periodontal homeostasis is not yet adequately studied. The presence of these species in higher values compared to the red complex could be clinically manifested as the absence of tooth loss and severe clinical attachment loss. The additional five group of genera identified in this study included *Odoribacter, Johnsonella*, *Oribacterium, Desulfobulbus*, and *Scardovia. Desulfobulbus* and *Johnsonella* genus were associated with subgingival plaque of periodontitis.^16^ The relative abundance of these genera represented a lower count, and their precise role in periodontitis are unidentified. Nevertheless, their detection has been associated with periodontitis in subgingival plaque. *Odoribacter* genus, within the family ‘Porphyromonadaceae’ are anaerobic and Gram-negative – which induced alveolar bone loss in a experiment of periodontal disease with rats.^36^ *Oribacterium* spp., are strictly anaerobic and Gram-positive species and it has been isolated from human subgingival dental plaque.^37^

The species belonging to *Leptotrichia* and *Selenomonas* genera – representing a higher relative abundance –were neither classified as health species nor pathogens as their role in either situation remains uncertain. *Leptotrichia* spp. are associated with both disease and normal health, representing distinct pathogenic potentials of bacteria in the same genera.^16^ *Selenomonas* species have been strongly associated with periodontitis, and they play a role in the onset and progression of the disease. Higher levels of *Selenomonas Sputigena* were detected in the subgingival biofilm of deep periodontal pockets ≤ 6mm, that represents Stage II & III periodontitis.^38^ However, their exact role in inducing periodontal destruction remains undetected, a higher prevalence of *Selenomonas* species identified in this study directly associates these species’ presence in the periodontal pocket of Stage II generalized periodontitis.

Apart from the role of periodontal microbes, it is essential to consider the host-microbial interaction for the development and manifestation of periodontitis. According to the ecological plaque hypothesis, the host's inflammatory responses and not the specific bacteria and putative virulence factor determines periodontitis development and progression.^39^ Moreover, the nutrient profile of the gingival crevice due to Gingival Cervicular Fluid (GCF) secretion produces a shift in the balance of resident microflora, leading to increased plaque biomass and high levels of obligate anaerobic Gram-negative bacteria.^40^ Thus the commensal flora would change to an opportunistic pathogenic flora by complex changes in the local environment, which are primarily driven by the host and not by the bacteria. However, it is noteworthy that the role of subgingival microflora in the etiology and pathogenesis of periodontitis is not diminished.

The beta-diversity measurement based on Unweighted UniFrac distance indicated that the distance between two different age groups was minimal. Although a total variance of 39.19% was observed for sex, the findings were not statistically significant. While bacterial species variation occurs in males and females, a non-significant difference in this study could have resulted from the exclusion of subjects who presented systemic conditions. Systemic conditions like pregnancy would induce hormonal changes and variation in bacterial species compared to male subjects. A Jaccard Index of 89%, similar in both age and sex, suggests that the microbial profile did not vary between biofilm samples and are representative of Stage II generalized periodontitis.

The 16s rRNA based microbial profiling via NGS in periodontal conditions is the first of its kind to be studied in the United Arab Emirates. Exact quantification of bacterial species less than 0.01% has not been described in our study, which could be a limitation. Also,16–23S rRNA providing high-resolution analyses for bacterial species identification has not been employed in our study. However, 16S rRNA-based bacterial profiling via next-generation sequencing of supragingival and subgingival plaque has been employed in previous studies to determine the composition and diversity of bacterial communities.^16^^,^^25^ Based on our study, it is possible to suggest that there is an association between Stage II generalized periodontitis and novel periodontal pathogens. Future studies based on proteomic and metabolomic analyses would provide information on the interbacterial relationship of these novel periodontal pathogens and improve our understanding on microbial function in periodontal pathogenesis.

## Conclusion

The 16S rRNA via NGS approach in patients diagnosed with Stage II generalized periodontitis indicated that the bacterial species belonging to the orange-complex and novel periodontal pathogens are the most abundant.
